# Hi-MDTCN: Hierarchical Multi-Scale Dilated Temporal Convolutional Network for Tool Condition Monitoring

**DOI:** 10.3390/s25247603

**Published:** 2025-12-15

**Authors:** Anying Chai, Zhaobo Fang, Mengjia Lian, Ping Huang, Chenyang Guo, Wanda Yin, Lei Wang, Enqiu He, Siwen Li

**Affiliations:** 1 College of Information Science and Engineering, Shenyang University of Technology, Shenyang 110870, China; chaianying@sut.edu.cn (A.C.); fangzhaobo2023@gmail.com (Z.F.); huangping0809@sut.edu.cn (P.H.); guo_cy@smail.sut.edu.cn (C.G.); yinwanda20@gmail.com (W.Y.); wanglei20250901@gmail.com (L.W.); lisiwen@sut.edu.cn (S.L.); 2 School of Mathematics and Information Engineering, Longyan University, No. 1 Dongxiao North Road, Xinluo District, Longyan 364012, China; 3 School of Chemical Equipment, Shenyang University of Technology, No. 30, Guanghua Street, Hongwei District, Liaoyang 111003, China; heq12345@gmail.com

**Keywords:** tool condition monitoring, multi-modal, multi-sensor fusion, deep learning

## Abstract

Accurate identification of tool wear conditions is of great significance for extending tool life, ensuring processing quality, and improving production efficiency. Current research shows that signals collected by a single sensor have limited dimensions and cannot comprehensively capture the degradation process of tool wear, while multi-sensor fusion recognition methods cannot effectively handle the complementarity and redundancy between heterogeneous sensor data in feature extraction and fusion. To address these issues, this paper proposes Hi-MDTCN (Hierarchical Multi-scale Dilated Temporal Convolutional Network). In the network, we propose a hierarchical signal analysis framework that processes the signal in segments. When processing intra-segment signals, we design a Multi-channel one-dimensional convolutional network with attention mechanism to capture local wear features at different time scales and fuse them into a unified representation. When processing signal segments, we design a Bi-TCN module to further capture long-term dependencies in wear evolution, mining the overall trend of tool wear over time. Hi-MDTCN adopts a dilated convolution mechanism, which can achieve an extremely large receptive field without building an overly deep network structure, effectively solving problems faced by recurrent neural networks in long sequence modeling such as gradient vanishing, low training efficiency, and poor parallel computing capability, achieving efficient parallel capture of long-range dependencies in time series. Finally, the proposed method is applied to the PHM2010 milling data. Experimental results show that the model’s tool condition recognition accuracy is higher than traditional methods, demonstrating its effectiveness for practical applications.

## 1. Introduction

Milling, as an efficient cutting process, utilizes rotating milling cutters to perform intermittent cutting on workpieces, capable of machining planes, grooves, threads, and complex geometric components, and is widely applied in industrial manufacturing and mass production [1]. Cutting tools are essential components in milling processes, and tool breakage can easily cause unexpected downtime of milling equipment. Accumulated tool wear leads to breakage, resulting in time and cost losses, with tool failures accounting for 7–20% of milling machine downtime and tool replacement costs accounting for 3–12% of total processing costs [2]. Although preventive replacement of milling tools is performed before breakage, replacement strategies rely on operator experience and lack accuracy. Research shows that the effective life of milling tools is typically only utilized at 50–80%; therefore, real-time monitoring of tool conditions can maximize tool life utilization and save processing costs [3].

As artificial intelligence advances, monitoring systems have gradually been applied to tool condition monitoring. Tool Condition Monitoring (TCM) systems are divided into offline and online types. Offline TCM systems require interruption of the machining process and use optical microscopes to inspect tool health during downtime periods [4]. Downtime inspection adds additional labor and time costs, and due to limited inspection frequency, it cannot timely detect tool wear changes. In contrast, in online TCM systems, machining operations continue without interruption, and data can be processed and tool conditions monitored in real time within specified cycles [5].

At present, techniques for identifying tool health status are divided into two categories: physics-based models and data-driven models. Physics-based models rely on physical laws or semi-empirical formulas to predict tool wear. These methods usually depend on mathematical descriptions of cutting physics, but due to the complexity and high nonlinearity of the cutting process, such methods are difficult to adapt to real-world conditions and have limited prediction accuracy [6]. In contrast, data-driven models can be trained with sufficient historical data and model the nonlinear dependencies between features extracted from sensor signals and cutting conditions to indirectly infer tool wear. Compared with physics-based models, data-driven models perform better in handling complex working conditions and large-scale data, and can achieve real-time monitoring based on sensor data. Therefore, data-driven models demonstrate broad application prospects in the field of tool wear monitoring [7]. However, current data-driven models struggle to capture both historical and future context in temporal data, and when facing multi-source sensor data, their ability to extract key wear features is limited, resulting in insufficient robustness under complex working conditions and making it difficult to further improve recognition accuracy.

To address these issues, this paper proposes a hierarchical multi-scale dilated temporal convolutional network (Hi-MDTCN) for multi-sensor fusion-based online TCM method. It employs a parallel branch structure to separately perform convolutional extraction and channel attention weighting on multi-source signals, significantly enhancing the representation quality of various types of signal features; secondly, it models temporal dependencies between segments through multi-scale dilated convolutional modules and combines residual connection mechanisms to enhance deep feature transmission capabilities; furthermore, it proposes a lightweight channel attention mechanism for dynamic weighting of intrasegment and intersegment features, effectively improving the model’s sensitivity to key wear features. Compared with traditional methods, Hi-MDTCN possesses higher parallel computing capability and stronger modeling stability, allowing the training complexity to be reduced while maintaining high prediction accuracy, thereby achieving the purpose of monitoring tool conditions in real time. The contributions of this paper are as follows

(a) This paper proposes a Bidirectional Temporal Convolutional Module (Bi-TCN), which employs a symmetric padding strategy to achieve non-causal modeling, enabling feature extraction to simultaneously capture both historical and future contextual information. Compared to the causal relationship constraints of traditional TCN, this module significantly enhances the temporal feature expression capability.
(b) For multi-source sensor data feature extraction, a lightweight intra-segment attention mechanism is designed, dynamically enhancing key wear features through channel attention weights. This mechanism can improve feature expression capability without increasing model complexity, effectively enhancing model robustness under complex working conditions.
(c) The paper proposes a Hierarchical Multi-scale Temporal Network Hi-MDTCN, and designs a two-stage hierarchical processing strategy for intra-segment and inter-segment in the network. Intra-segment processing employs three-way parallel convolutions to process multi-source signals and introduces channel attention mechanisms to strengthen key features. Inter-segment processing adopts multi-scale dilated convolutional mechanisms to capture multi-scale patterns in the tool wear evolution process. The architecture achieves collaborative modeling of local features and global trends through the integration of time series slices.

The remaining parts are organized as follows. Section 2 reviews some related work on current TCM methods, Section 3 briefly introduces the basic theory related to TCN, Section 4 elaborates in detail the proposed Hi-MDTCN method framework. Section 5 demonstrates the effectiveness of the proposed method in tool milling datasets and compares it with other methods. Finally, Section 5 provides the conclusion.

## 2. Related Work

Deploying TCM systems can effectively improve production quality and reduce energy consumption [8]. At present, data-driven TCM methods are divided into direct methods and indirect methods [9]. Direct methods measure tool geometry based on vision systems; however, due to the high cost of high-speed cameras and the large amount of data generated that require substantial computational resources to process, the predictability of online monitoring processes is usually lower [10].

Indirect sensing methods determine tool wear by analysing multi-source signals, enabling tool condition monitoring with minimal interference during actual cutting processes [11]. Compared to direct methods, indirect methods are more practical and cost-effective [12,13]. However, indirect methods are typically related to the machine tool’s response to the cutting process, are susceptible to noise interference, and therefore often less accurate than direct methods [14,15]. TCM using indirect methods relies on traditional signal processing techniques, and due to the highly non-linear and multi-variate coupling characteristics of the cutting processes, it is difficult to extract effective features from complex multi-source sensor data [16,17].

In recent years, as artificial intelligence advances rapidly, machine learning approaches have substantially enhanced tool condition recognition accuracy in indirect monitoring methods by leveraging automated feature extraction and pattern recognition [18,19,20]. Machine learning algorithms such as Support Vector Machine (SVM) [21], Random Forest (RF) [22], and K-Nearest Neighbor (KNN) [23] have been used for tool wear prediction, but these methods still rely on manual feature engineering and have limited expression capability for high-dimensional data. To address these limitations, deep learning techniques have increasingly emerged as research focal points owing to their robust automatic feature extraction capabilities and superior performance in handling complex data [24,25]. Zhu et al. [26] utilized fast neural network methods to extract features from cutting force signals, achieving effective monitoring of tool wear in micro-milling processes. Huang et al. [27] proposed a tool wear monitoring method combining Short-Time Fourier Transform with deep convolutional neural networks, implementing adaptive feature extraction and wear state prediction by modeling the time-frequency characteristics of vibration signals. Wang et al. [28] extracted time-frequency features of acoustic emission signals through Fourier transform and wavelet packet decomposition, and used BP neural networks for modeling and analysis; experimental results showed that the tool wear monitoring method based on BP neural networks outperformed SVM. Although the above studies have confirmed the effectiveness of deep learning for tool condition monitoring, they only analyze single-source signals. Single sensor signals often can only reflect certain aspects of tool conditions and struggle to comprehensively cover the actual working conditions of tools in complex machining processes.

Compared to single-source signal monitoring methods, multi-sensor fusion technology can significantly improve the accuracy and robustness of tool wear prediction, reducing the impact of environmental interference and signal uncertainty [29,30]. Li et al. [31] proposed a residual variational autoencoder transfer learning model based on multi-sensor fusion, optimising the encoding structure of variational autoencoders through residual networks to achieve fault diagnosis between different tools under parameter transfer strategies. Lin et al. [32] effectively improved the accuracy of tool wear state recognition by establishing Stacked Sparse Autoencoders (SSAE) and BP neural networks for multi-sensor signals, respectively. Song et al. [33] proposed a tool wear monitoring method that fuses multi-kernel Gaussian process regression with stacked multi-layer denoising autoencoders, achieving high precision feature representation learning and reliable wear state prediction by optimising kernel function parameters and denoising force signal features. The above autoencoder-based methods can effectively extract deep features from multi-source sensor data and enhance feature representation capabilities through optimisation mechanisms such as residual networks and stacked sparse structures, providing feasible solutions for tool condition monitoring in actual machining processes. However, traditional AE structures struggle to capture the temporal changes during the tool wear process and cannot effectively model the dynamic process of wear evolution.

To address this issue, recurrent neural network-based methods have become a new research direction. Recurrent neural networks, through memory units and gating mechanisms, can better preserve historical information and temporal dependencies of tool wear, further improving the accuracy of monitoring and real-time performance. Sayyad et al. [34] combined LSTM with feature selection to optimize and sequentially model sensor signals, providing reliable monitoring means for tool wear control. Zhou et al. [35] designed a dual parallel BiLSTM architecture to process the multi-domain features of vibration and cutting force signals separately, achieving an accurate prediction of tool wear states with temporal dependency patterns. Xiao et al. [36] proposed a tool wear prediction method based on Bayesian optimized Gated Recurrent Units (GRU), achieving wear state prediction with high generalization capability by modeling time domain, frequency domain, and time-frequency domain features of multi-working condition signals with hyperparameter optimization. Liu et al. [37] effectively improved the accuracy of tool wear state recognition by deep mining temporal feature dependencies through BiGRU networks combined with variational mode decomposition for signal optimisation processing. Although RNNs can effectively model the dynamic evolution process of tool wear, their current state prediction must be based on the calculation results of the previous moment, causing errors to accumulate during temporal propagation, leading to a decrease in the prediction accuracy as time steps increase [38].

To overcome these problems, one-dimensional convolutional neural networks (1D-CNN), with their local receptive fields and parameter-sharing mechanisms, can efficiently extract local patterns and features from temporal data while reducing computational complexity. Wang et al. [39] proposed a multi-sensor tool wear diagnosis method combining 1D-CNN with Deep Generalized Canonical Correlation Analysis (DGCCA), achieving accurate prediction of tool wear states by using DGCCA with attention mechanisms to fuse output features from various networks and remove redundant information. Dong et al. [40] proposed an improved CaAt-ResNet-1d model, effectively enhancing the accuracy of tool wear states in complex cutting environments by adding CaAt1 channel attention mechanisms and CaAt5 mechanisms for automatically learning features of different channels in residual network blocks. Yang et al. [41] significantly improved the stability of tool wear state prediction under varying working conditions by using multi-scale entropy values as conditional indicators to construct one-dimensional feature vectors for input into 1D CNN. Furthermore, by converting temporal signals into two-dimensional image representations, two-dimensional convolutional neural networks (2D-CNN) can simultaneously capture spatial structural features in the time-frequency domain, providing more comprehensive feature expression capabilities for tool wear state monitoring and further improving model prediction accuracy. Zhang et al. [42] enhanced the model’s capability to identify various tool wear stages by transforming force signals into two-dimensional image formats using time-frequency analysis methods and implementing convolution kernels with varying sizes to capture multi-scale features simultaneously. Li et al. [43] proposed an integrated convolutional neural network architecture, achieving real-time monitoring of tool conditions during cutting processes by combining Short-Time Fourier Transform techniques to map one-dimensional acoustic signals to two-dimensional representations in the time-frequency domain. Liu et al. [44] proposed a real-time milling chatter detection method based on Continuous Wavelet Transform (CWT) scalograms and deep convolutional neural networks (CNN), providing a reliable solution for real-time monitoring of tool wear in milling processes. Although CNNs have achieved good performance in tool wear monitoring, there are still some limitations. For example, CNNs often use fixed-size convolution kernels, resulting in limited receptive fields and difficulties in modeling long-distance dependencies [45]. Furthermore, extracting features from the time and frequency domain from multi-sensors increases model complexity; CNN channels need to share convolution kernels, and excessive information exacerbates the difficulty of extracting and fusing redundant features, easily introducing unnecessary information and affecting the quality of the feature [46]. When using 2D-CNN for tool wear monitoring, temporal signals need to be converted to time-frequency images as input, with preprocessing steps that are computationally expensive and complex, bringing additional computational overhead to the model and introducing extra delay [47]. Therefore, 2D-CNN methods are often not efficient enough in tool monitoring applications with high real-time requirements.

To compensate for the shortcomings of the above research methods, the Hi-MDTCN proposed in this paper breaks through the limitations of traditional methods through its hierarchical design and bidirectional temporal convolutional mechanism. Its advantages lie in the ability to simultaneously capture contextual information from both past and future, adaptively filter important features through lightweight channel attention mechanisms, and implement long dependency relationship modeling using multi-scale dilated convolutions. This architecture does not require complex signal conversion preprocessing, directly processes original temporal data, significantly reduces computational delay while maintaining high prediction accuracy and generalization capability, providing a real-time reliable solution for real-time tool condition monitoring.

## 3. Preliminaries

During the tool wear process, the evolution of wear states exhibits strong historical dependence, with the current evolution significantly influenced by previous processing conditions. This temporal evolution characteristic can be formally expressed as Equation (1):

(1) $$s_{t}=f\left(s_{t-1},s_{t-2},\ldots ,s_{t-n}\right)+\epsilon_{t}$$

where $s_{t}$ is the tool wear state at time *t*, and $\epsilon_{t}$ is random noise.

Therefore, TCM is essentially a sequence modeling problem, which requires the capture of temporal dependencies to achieve accurate wear prediction and optimize tool replacement strategies and production efficiency. TCN addresses the sequence modeling problem by ensuring temporal causality through causal convolutions, meaning current predictions rely only on historical data, avoiding future information leakage. Simultaneously, it employs dilated convolutions to exponentially expand the receptive field, efficiently capturing long-term evolution patterns through sparse sampling. These architectural characteristics enable TCN to possess both local feature extraction and global trend modeling capabilities, providing an effective solution for complex time series modeling problems in tool condition monitoring.

### 3.1. Causal Convolutions

In the time series modeling domain, causal convolution is a core component of TCN. This structure strictly enforces temporal causality constraints, meaning that the output at the current time step can only depend on input information from the current and previous time steps, and cannot access data from future time steps. Its mathematical expression is given by Equation (2).

(2) $$y_{t}=\sum_{i=0}^{k-1}w_{i}\cdot x_{t-i}$$

where, $y_{t}$ represents the output feature at time step *t*, $w_{i}$ represents the *i*-th weight parameter of the convolution kernel, *k* represents the size of the convolution kernel in the time dimension, and $x_{t-i}$ represents the input feature at relative position *i*. This approach ensures the model cannot access future information, satisfying real-time prediction requirements.

### 3.2. Dilated Convolution

**Figure 1:**
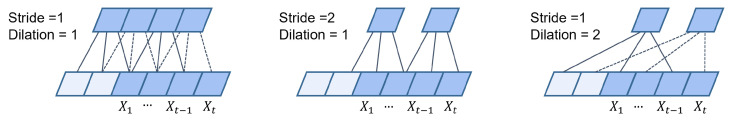
Shows examples of TCN combining causal convolution and atrous convolution at different values of stride and atrous rate, applying a 1d convolution with kernel sizes k = [3; 3; 3], dilation d = [1; 1; 2] and strides s = [2; 1; 1].

The historical information capacity of causal convolution only increases linearly with network depth, making it difficult to process sequence tasks requiring long-term dependencies. Dilated convolution, as the second core component of TCN, enhances the network’s modeling capability for long sequence dependencies by introducing sampling strategies with gaps. In contrast to standard causal convolution, dilated convolution can exponentially increase the receptive field while preserving parameter efficiency, successfully addressing the difficulty of capturing long-term dependencies. For 1-D sequence input $x\in R^{n}$, and filter f:{0,⋯,k−1}→R, the dilated convolution operation *F* on sequence element *s* is defined by Equation (3):

(3) $$F\left(s\right)=\left(x{*}_{d}f\right)\left(s\right)=\sum_{i=0}^{k-1}f\left(i\right)\cdot x_{s-d\cdot i}$$

where *d* is the dilation factor, *k* is the filter size, and s−d·i represents the direction toward past time steps. Therefore, dilated convolution is equivalent to introducing a fixed step length between each tap of the neighboring filter. When d=1, dilated convolution reduces to regular convolution. Using larger dilation factors enables perception of inputs from more distant positions, effectively expanding the receptive field of the convolutional neural network. The TCN combined with causal dilated convolution is shown in Figure 1.

## 4. Methodology and Proposed Model

**Figure 2:**
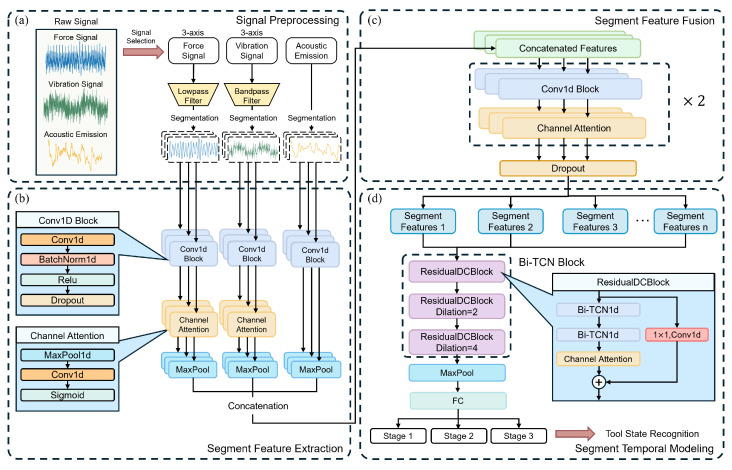
Framework of the proposed approach. (**a**) illustrates signal preprocessing, including filtering and segmentation. (**b**,**c**) represent segment feature extraction and fusion, enhancing intra-segment features. (**d**) shows temporal modeling across segments to monitor tool condition.

The proposed Hi-MDTCN architecture consists of three stages: signal preprocessing, intra-segment feature extraction, and inter-segment temporal modeling. The specific framework is shown in Figure 2.

First, in the signal preprocessing stage, a Butterworth filtering strategy is adopted for different types of sensor signals to eliminate noise and extract representative features. Next, in the intra-segment feature extraction stage, a Bi-TCN block is introduced, which employs a symmetric padding strategy to achieve non-causal modeling. This allows feature extraction to simultaneously capture both historical and future contextual information, significantly enhancing the temporal feature representation capability compared to the causality constraints of traditional TCNs. Meanwhile, for the feature extraction of multi-source sensor data, a lightweight intra-segment attention mechanism is designed, which dynamically enhances key wear features through channel attention weights, improving feature representation capability without increasing model complexity, and effectively enhancing the robustness of the model under complex working conditions. Finally, in the inter-segment temporal modeling stage, we propose a hierarchical multi-scale temporal network architecture, which achieves an exponentially expanded receptive field by designing combinations of dilated convolutional layers with increasing dilation rates, thereby enabling efficient and parallel capture of long-term temporal dependencies. The proposed model adopts a hierarchical processing strategy that first extracts local intra-segment features and then aggregates them into global representations.

This approach addresses the issues of gradient vanishing and inefficient parallel computation in traditional recurrent structures for long sequence modeling, while also avoiding the computational overhead of complex time-frequency transformations required by 2D-CNN methods. As a result, the model achieves better training stability and inference capability while maintaining high prediction accuracy.

### 4.1. Signal Preprocessing

By analyzing the first-order difference features of the force sensor signal, the end time of the feed and the start time of the tool retraction during the milling process can be detected, enabling precise extraction of the effective cutting segment. The original force signal usually contains high-frequency noise, which can be effectively reduced by applying a moving average window for smoothing. Mathematically, this is expressed as Equation (4)

(4) $$f_{\mathrm{smooth}}\left[i\right]=\frac{1}{W}\sum_{j=i-\frac{W-1}{2}}^{i+\frac{W-1}{2}}f_{\mathrm{raw}}\left[j\right]$$

where $f_{\mathrm{raw}}\left[j\right]$ denotes the amplitude of the original force signal at sampling point *j*, and $f_{\mathrm{smooth}}\left[i\right]$ denotes the amplitude of the smoothed signal at sampling point *i*. *W* represents the width of the moving average window, and the summation range for *j* is from $i-\frac{W-1}{2}$ to $i+\frac{W-1}{2}$, with the window centered at the current point.

To accurately detect feed end and retraction start, the force signal is smoothed to suppress high-frequency noise while preserving transient trends. The moving-average window size *W* is defined as Equation (5)

(5) $$W=\left\lfloor \frac{f_{s}\times 60}{k\times n\times Z}\right\rceil ,$$

where $f_{s}$ is the sampling frequency (Hz), *n* is the spindle speed (rpm), and *Z* is the number of tool teeth. The parameter *k* denotes the smoothing coefficient, and ⌊·⌉ indicates rounding toward the nearest integer with a lower bound. This formulation exploits the physical periodicity of the tooth-passing frequency $f_{\mathrm{TPF}}=n\times Z/60$. The window should span multiple tooth-passing periods to suppress periodic impacts and harmonics while avoiding excessive smoothing that would blur transients. Here, k∈[0.1, 0.3] corresponds to the number of tooth-passing periods covered by the window, balancing noise suppression and transient preservation.

After smoothing, a first-order difference is applied to calculate the difference between adjacent sampling points, thereby detecting abrupt changes in the signal. Mathematically, this is expressed as Equation (6)

(6) $$diff\left[i\right]=f_{\mathrm{smooth}}\left[i+1\right]-f_{\mathrm{smooth}}\left[i\right]$$

where diff[i] denotes the first-order difference at sampling point *i*, and $f_{\mathrm{smooth}}\left[i\right]$ and $f_{\mathrm{smooth}}\left[i+1\right]$ denote the amplitudes of the smoothed signal at two adjacent sampling points.

A positive value indicates that the signal is rising, which may correspond to the end of the tool feed, with the tool entering a stable cutting state. A negative value indicates that the signal is falling, which may correspond to the start of tool retraction.

Based on the statistical characteristics of the difference, the weighted sum of the mean and standard deviation of the absolute value of the difference sequence is used as the threshold to ensure good adaptability for signals of different strengths, as shown in Equation (7):

(7) $$\theta =\bar{d}+k\cdot \sigma_{\left|d\right|}$$

here, θ is the threshold, $\bar{d}$ represents the mean of the difference sequence, *k* is an adjustable weighting coefficient, and $\sigma_{\left|d\right|}$ is the standard deviation of the absolute value of the difference sequence. Based on this threshold, continuous running windows are used to detect the start and end points. When the absolute value of the difference in the window is consistently greater than the threshold and the sign is consistent, it is determined as the end of the feed or the start of tool retraction. The signal segment near the end of the feed or the start of tool retraction reflects the state characteristics of the tool approaching the wear limit. To further enhance the effective signal features, it is necessary to filter out noise and remove interference from high-frequency noise in the signal segment.

The filtering method is selected according to the frequency characteristics of the signal, including low-pass, high-pass, and band-pass. For different types of signals, different filtering strategies are adopted to effectively remove noise and improve the accuracy of feature extraction. Therefore, a Butterworth filter is designed to perform low-pass filtering, retaining the low-frequency components of the signal and smoothing the signal. The transfer function of an *n*-order Butterworth filter with cutoff frequency $\omega_{c}$ can be mathematically expressed as Equation (8):

(8) $$H\left(\omega \right)=\frac{1}{1+{\left(\frac{\omega }{\omega_{c}}\right)}^{2n}}$$

**Figure 3:**
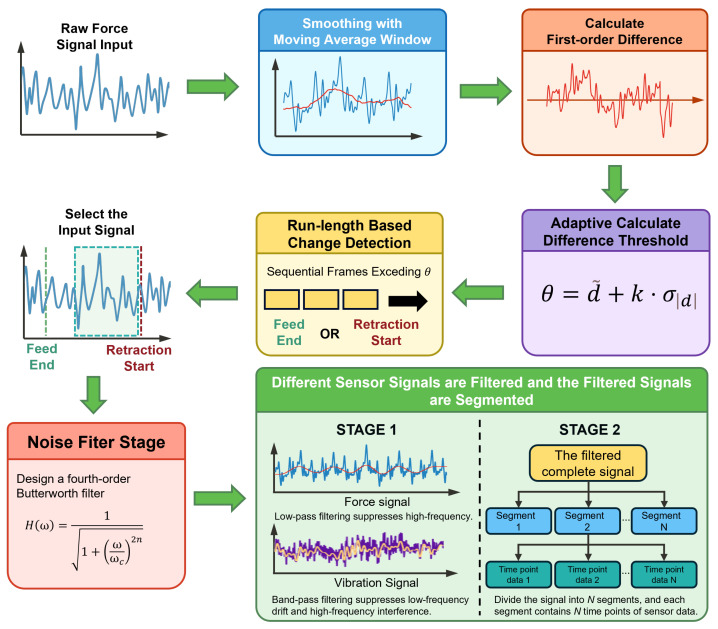
Signal preprocessing procedure.

Here, ω is the frequency, $\omega_{c}$ is the cutoff frequency, and *n* is the filter order. For force signals, low-pass filtering is used so that the low-frequency components of the signal can pass through without attenuation, while the high-frequency components are attenuated according to the filter order, suppressing high-frequency noise in the signal and vibration features. Vibration signals are processed with band-pass filtering, allowing the signal within a specific frequency band to pass through without attenuation, thereby suppressing drift and low-frequency noise, while retaining frequency bands related to vibration. The Butterworth filter has the characteristic of the maximum flatness in the passband among all types of filters, ensuring that the phase and amplitude of the signal are not distorted by filtering, as shown in Figure 3.

Traditional long-term sequence signal modeling methods have high computational complexity and memory usage, making them difficult to train. In addition, it is difficult for a single time step sampling point to directly represent the tool wear state; local feature extraction through continuous time windows is required to effectively reveal the wear evolution pattern. To address these issues, a hierarchical two-stage processing strategy is designed. By uniformly dividing the stable cutting segment into multiple time slices, multi-sensor fusion features are extracted for each short segment within the segment, and temporal associations between different segments are established to capture the wear evolution trend. The hierarchical architecture effectively solves the problem of exponential growth in computational complexity caused by direct modeling of long sequences; second, the complementary fusion of multi-segment features can significantly improve the robustness of the model to noise. In addition, parallel processing of each segment greatly improves computational efficiency. Through the hierarchical design of the time series, both local detail changes and global trend information are preserved, providing a more comprehensive and reliable basis for accurate monitoring of tool wear state.

### 4.2. Intra-Segment Feature Extraction

**Figure 4:**
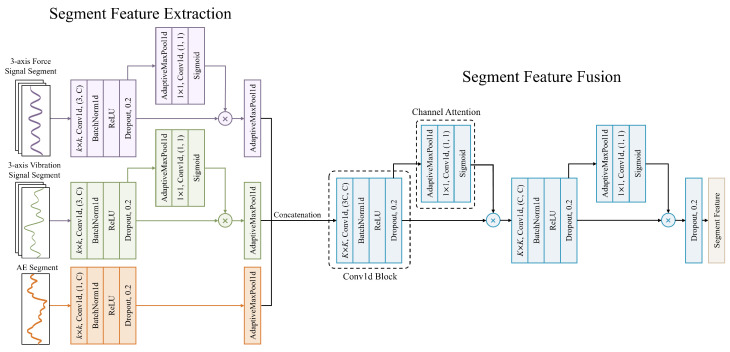
Intra-segment feature extraction process.

In order to capture short-term local features during the tool wear process, an intra-segment feature extractor is designed to extract features in parallel from signals of multiple sensors within each time segment. Specifically, after preprocessing and segmentation, the original multi-channel sensor signals are input into a three-branch parallel lightweight convolutional network, with each branch corresponding to one type of sensor signal: triaxial force sensing, triaxial vibration sensing, and acoustic emission sensing signals. Each branch adopts the same structure: first, a lightweight one-dimensional convolutional block is used to extract local temporal features, then a channel attention mechanism is applied to weight the features of each channel, and finally, global pooling is used to compress the temporal information into a feature vector. Although the number of channels of the original input needs to be increased during feature extraction, since the acoustic emission signal is single-channel at the input, the effect of setting a channel attention module is not obvious. Therefore, after feature extraction of the acoustic emission signal is completed, it is directly pooled and then concatenated with other features for fusion. The intra-segment feature extraction architecture is shown in Figure 4.

A lightweight one-dimensional convolutional module, Conv1dBlock, is used to extract the features of each branch. Each module contains a convolution kernel of length *k*, BN, ReLU and Dropout. For the $c_{m}$ channels, with input features $X\in R^{c_{m}\times L}$ of temporal length *L*, the output at channel *j* and time *t* is given by Equation (9):

(9) $$y_{j,t}=\sum_{i=1}^{c_{m}}\sum_{k=0}^{K-1}w_{j,i,k}\;x_{i,t-k}+b_{j}$$

here, $w_{j,i,k}$ are the convolution kernel parameters, and $b_{j}$ is the bias term. After convolution, batch normalization and ReLU activation are applied, followed by Dropout, which can effectively extract local temporal features and suppress overfitting. After feature extraction by the one-dimensional convolutional module, the output features are input into the channel attention module to focus on important channel information. The attention module first performs global pooling on each channel to obtain the channel description vector $z,\;z\in R^{c}$, and then passes it through a one-dimensional convolution and sigmoid function to calculate the channel weights, as mathematically expressed in Equation (10):

(10) $$\left\{\begin{array}{cc} \; & \alpha =\sigma (f_{1D}\left(z\right))\\ \; & \sigma \left(x\right)=\frac{1}{1+e^{-x}} \end{array}\right.$$

Here, $f_{1D}$ denotes a learnable 1D convolution operation with kernel size *k*, and σ(·) is the Sigmoid activation function. Specifically, for the *c*-th channel, as shown in Equation (11):

(11) $$\left\{\begin{array}{cc} \; & \alpha_{c}=\sigma \left(\sum_{j=-\frac{k-1}{2}}^{\frac{k-1}{2}}u_{j}\;z_{c+j}\right)\\ \; & {\hat{x}}_{c,t}=\alpha_{c}\;x_{c,t} \end{array}\right.$$

Here, $u_{j}$ is the 1D convolution kernel parameter, $\alpha_{c}\in \left(0,\;1\right)$ is the attention weight of channel *c* after attention weighting. The channel attention mechanism can achieve cross-channel interaction through local convolution, and introducing a small number of parameters can significantly improve the effectiveness of channel attention. After attention weighting, the temporal feature maps of each branch highlight the key channel information. For each branch, after global pooling and attention weighting, the temporal feature map is compressed to a feature vector of length *C* by max pooling. For example, if the output feature dimension is C×L, it is pooled to $R^{C}$, where each element can be used as the maximum response of the corresponding channel. The feature vectors output by the force sensing branch, vibration sensing branch, and acoustic emission branch are denoted as $f_{dyn},f_{acc},f_{ae}\in R^{C}$. After obtaining the feature vectors of each branch, the three branch features are concatenated along the channel dimension to form a feature vector of length 3C, $f=\left[f_{dyn};f_{acc};f_{ae}\right]\in R^{3C}$. In order to further integrate multi-source information and enhance joint features, the features extracted by the three parallel convolution branches are concatenated, and then input into a fusion convolution layer. The feature vector concatenated after the first Conv1dBlock and its attention layer is reshaped to a tensor of size 3C×1, which is input into a convolution kernel of size *K* in the Conv1dBlock. This fusion convolution reduces the number of channels from 3C to *C*, followed by an attention layer to reweight the fused features. Then, a second Conv1dBlock and its attention layer are used for further feature integration, and finally, a fused feature vector of length *C* is output. The entire process can be regarded as first performing feature extraction and weighting at the branch level, and then obtaining a unified representation through convolution and fusion at the segment level. Finally, a Dropout layer is added at the output to prevent overfitting.

The design of parallel branches enables the network to extract its own key patterns for different types of signals, while also improving computational efficiency. The parallel convolutional structure and attention weighting of multi-source signals significantly enhance the expressive power of intra-segment features and the quality of multi-modal information fusion, providing a richer and more reliable representation for subsequent temporal modeling.

### 4.3. Inter-Segment Temporal Modeling

According to the regulations of numerical control machining programs, tool changes must be performed at the safe and fixed position of the machine tool. Therefore, the judgment of tool status needs to be based on the comprehensive information of the machining stage signals, rather than relying solely on the local signal at a certain moment. Therefore, we propose the design of Bi-TCN, which can utilize both the past and future temporal information within the current stage, instead of only using past information like TCN.

**Figure 5:**
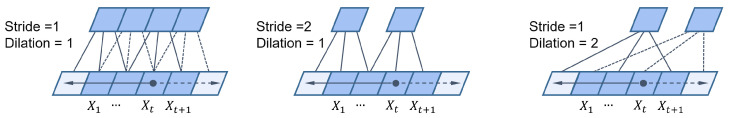
Shows examples of Bi-TCN1d combining causal convolution and atrous convolution at different values of stride and atrous rate, applying a 1d convolution with kernel sizes k = [3; 3; 3], dilation d = [1; 1; 2] and strides s = [2; 1; 1].

Therefore, in the inter-segment temporal modeling stage, a Bi-TCN1d layer is designed to capture long-term dependencies between different time segments. The forward convolution branch processes the past-direction information of the time series, while the backward convolution branch processes the future-direction information. In this way, the output of the Bi-TCN1d layer at each moment can take into account the contextual dependencies before and after that moment, achieving bidirectional temporal modeling, as shown in Figure 5.

**Figure 6:**
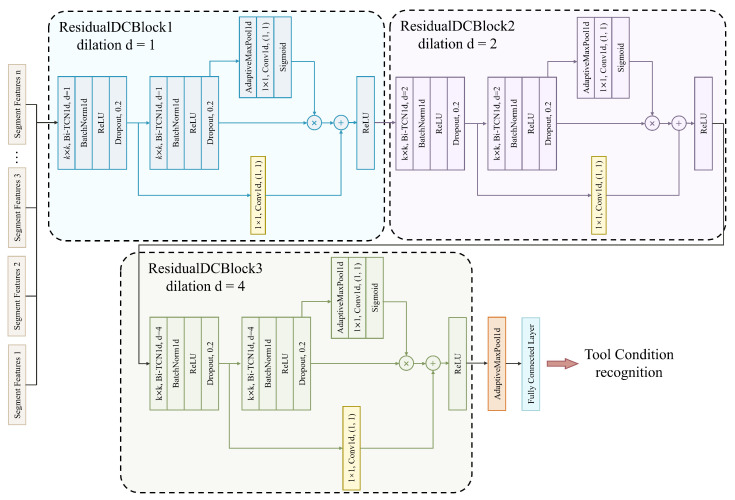
Inter-segment temporal modeling process for identifying tool states. The Bi-TCN Block consists of three stacked ResidualDCBlocks with dilation rates of 1, 2, and 4.

At the same time, we adopt a multi-layer stacked residual dilated convolutional network (Residual Dilated Convolutional Block, ResidualDCBlock) to capture long-term dependencies between different time segments. Each ResidualDCBlock module contains two consecutive Bi-TCN1d layers. By using symmetric padding, the output sequence length is kept the same as the input, achieving non-causal convolution. After each convolution, BN, ReLU activation, and dropout are applied in sequence to enhance the stability and robustness of feature extraction. The overall process is shown in Figure 6.

**Figure 7:**
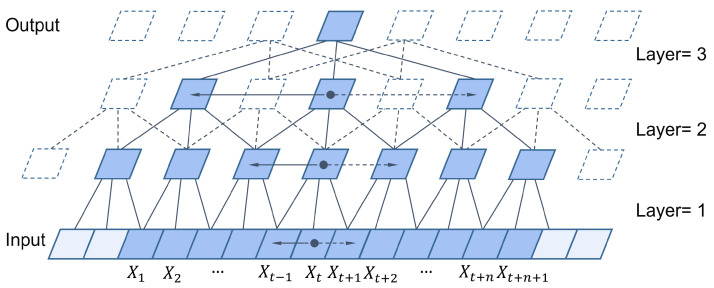
The Bi-TCN module with exponentially expanding receptive fields through stacked layers of different dilation rates. Shown are three Bi-TCN1d layers with kernel sizes k = [3; 3; 3], dilation rates d = [1; 1; 2] and strides s = [2; 1; 1].

The first Bi-TCN1d first performs feature transformation on the input sequence, and the output is passed through a second Bi-TCN1d layer with the same configuration, then enters the channel attention module, where the features from the forward and backward branches are fused. The attention module performs global pooling on the output features, followed by a 1×1 one-dimensional convolution and Sigmoid activation to generate channel attention weights, highlighting the channel information that more effectively reflects the tool wear state. The final output of the module is the result of the residual connection, that is, the sum of the output from the convolutional layer and the channel attention module with the input. If the number of output channels does not match, a 1×1 one-dimensional convolution is used to match the dimensions, and then ReLU activation is applied to obtain the final features. Specifically, the module takes the segment feature sequence $X\in R^{c_{m}\times L}$ as input, where $c_{m}$ is the number of input channels and *L* is the length of the sequence. The output of the module is defined by Equation (12):

(12) y=ReLU(F(x)+H(x))

here, F(x) represents the composite mapping in the residual block, including two layers of learnable convolution with dilation, batch normalization, ReLU, and dropout operations, followed by a channel attention module. H(x) is the identity mapping; when the number of input and output channels is inconsistent, a 1×1 convolution is introduced to match the dimensions, as mathematically expressed in Equation (13):

(13) $$H\left(x\right)=\left\{\begin{array}{cc} x, & c_{m}=c_{n}\\ {\mathrm{Conv}}_{1\times 1}\left(x\right), & c_{m}\neq c_{n} \end{array}\right.$$

here, $c_{n}$ is the number of output channels. The residual structure allows each module to directly “skip” the input signal to the output, thus enhancing the advantage of increasing the depth of the network in a hierarchical manner. In Bi-TCN, we stack multiple ResidualDCBlock layers, with each layer’s dilation rate increasing exponentially, which exponentially expands the receptive field of the network. Therefore, after *L* convolutional layers, the size of the final receptive field can be calculated similarly to Equation (14):

(14) $$R=\left(k-1\right)\left(2^{L}-1\right)+1$$

here, *k* is the kernel size of each layer, and *L* is the number of convolutional layers. By increasing the dilation rate, the network can maintain a wide temporal coverage with both shallow and deep layers, effectively obtaining global trend information across multiple segments, as shown in Figure 7.

The residual connections in each layer provide shortcuts for information propagation in deep networks, allowing gradients to flow directly across layers and greatly alleviating the common problem of gradient vanishing in deep networks. In addition, the attention module dynamically adjusts channel weights in each layer, so that key wear characteristics are enhanced during propagation, improving the model sensitivity and robustness to subtle signals.

Compared with traditional recurrent neural networks, Bi-TCN can compute the output at each position of the sequence in parallel. Therefore, when processing long sequences, Bi-TCN usually has faster training and inference speed. Parallelism and non-causality enable Bi-TCN to utilize both intra-segment and inter-segment information simultaneously. Thus, Bi-TCN not only inherits the parallelism and stability advantages of TCN, but also more fully integrates temporal context through its bidirectional design, achieving sequence modeling of multi-stage, non-stationary tool wear.

The overall description of the proposed method is shown in Algorithm A1 in Appendix A for details., including signal preprocessing (lines 1 to 7), intra-segment feature extraction (lines 8 to 15), and inter-segment temporal modeling (lines 16 to 20).

## 5. Experimental Study

We conducted experiments on the PHM2010 dataset to test the performance of the proposed method and verify its effectiveness. The code for this paper is available at: https://github.com/2023AIOT/Hi-MDTCN/, accessed on 11 December 2025.

### 5.1. Dataset Description

The PHM2010 dataset was collected under dry milling conditions using a high-speed CNC machine Roders Tech RFM760 throughout the entire tool life cycle, and contains a total of six tool life cycle experiments. Among them, three cutting experiments, C1, C4, and C6, recorded the tool wear value after each machining stage. The materials of the milling cutter and workpiece are ball-end tungsten carbide tool and stainless steel HRC52, respectively. The workpiece surface was machined row by row along the x-axis using a 6-mm three-flute cutter. After completing one stroke along the x-axis, the tool was retracted to start a new stroke. At the end of each stroke, the flank wear of the three tool teeth was measured offline using a LEICA MZ12 microscope. The specific experimental conditions are shown in Table 1.

**Figure 8:**
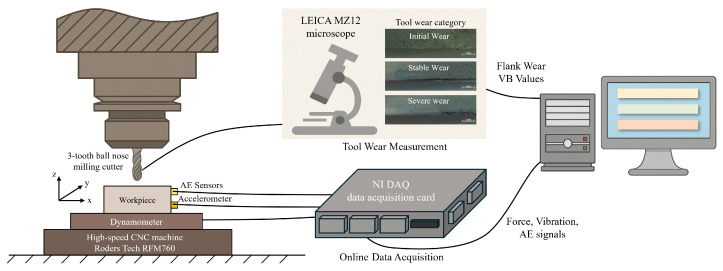
Experimental equipment and configuration.

During the milling process, a Kistler 9265B three-component dynamometer was installed between the worktable and the workpiece to collect force signals in the x, y, and z directions during the cutting process. At the same time, three Kistler 8636c acceleration sensors were directly fixed on the workpiece to measure the machine tool vibration data in the x, y and z directions, respectively. In addition, a Kistler AE sensor was installed on the workpiece to monitor high-frequency stress waves. All sensor signals were collected using an NI DAQ PCI1200 board with a sampling frequency of 50 kHz. Therefore, this dataset contains seven types of signals: force signals in the x, y, and z axes (N), vibration signals in the x, y and z axes (g), and the root mean square value of the acoustic emission signal AE-RMS (V). The experimental setup is shown in Figure 8.

### 5.2. Data Preprocessing

**Figure 9:**
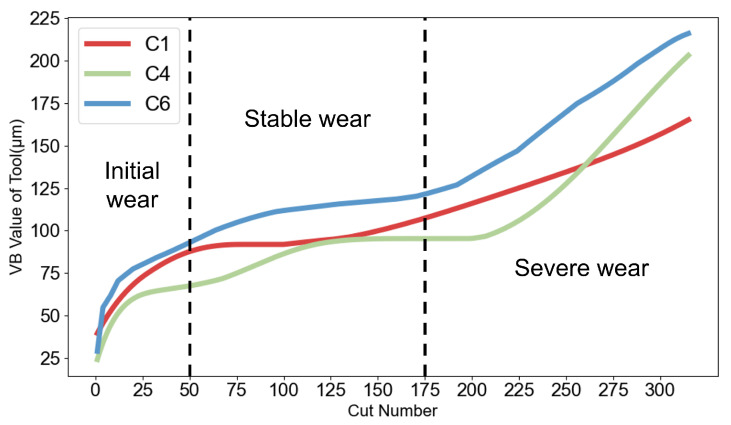
Experimental equipment and configuration.

Since experiments C1, C4, and C6 documented tool-wear measurements following each machining operation, these three experiments served as the dataset for this research. After each machining operation, three flank-wear values are recorded for the tool. In this study, the arithmetic mean of these three flank-wear measurements is taken as the final flank-wear value for that experimental record, and the tool-wear stages are evaluated based on the trend of these averaged wear values. The tool wear curves throughout the entire operational cycles of experiments C1, C4, and C6 are shown in Figure 9.

All cutting tools across the three experiments entered the stable wear phase following the 50th milling operation; nevertheless, the commencement timing of the severe wear phase varied across different experiments. Given that experiment C6 consistently exhibited higher tool wear levels, the initiation point of severe wear in experiment C6 served as the benchmark for differentiating between stable wear and severe wear stages. Consequently, tool wear data spanning operations 1–50 were categorized as initial wear, operations 51–175 as stable wear, and operations 176–315 as severe wear. Information from all seven sensor channels was integrated into the preprocessing workflow. To guarantee reliable assessment of model performance, the experimental dataset was partitioned into three groups for pairwise cross-validation, with comprehensive details presented in Table 2.

**Figure 10:**
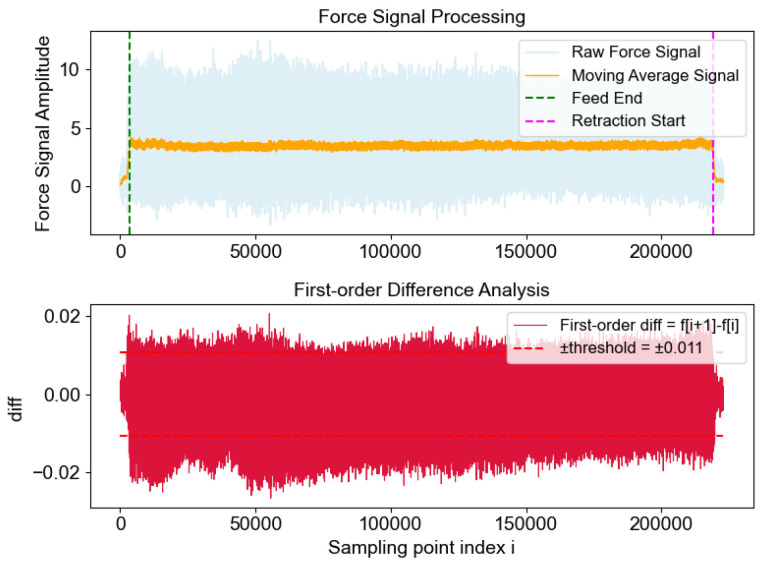
Force signal processing, analysis of the end of the feed and start position of the retraction.

The data acquisition card has a sampling frequency of up to 50 kHz, and each cutting test takes 15 to 30 s. For each cut, the data samples from each sensor, including the feed and retraction stages, contain more than 100,000 data points. Selecting data from the stable cutting stage close to the retraction phase best reflects the tool wear condition. By analyzing the first-order difference features of the force sensor signal, the specific positions of the end of the feed and the start of the retraction can be obtained, as shown in Figure 10. Finally, 50,000 sensor records generated during 1 second of stable cutting closest to the start of retraction were selected, and the original signals were filtered according to the filtering method described in Section 3.1.

The architectural parameters are detailed in Table 3. The parameter selection was designed based on comprehensive considerations of the feature dimensions of multi-sensor data, temporal dependency length, and computational efficiency. Experimental validation shows that this parameter configuration achieves favorable results in experiments.

**Figure 11:**
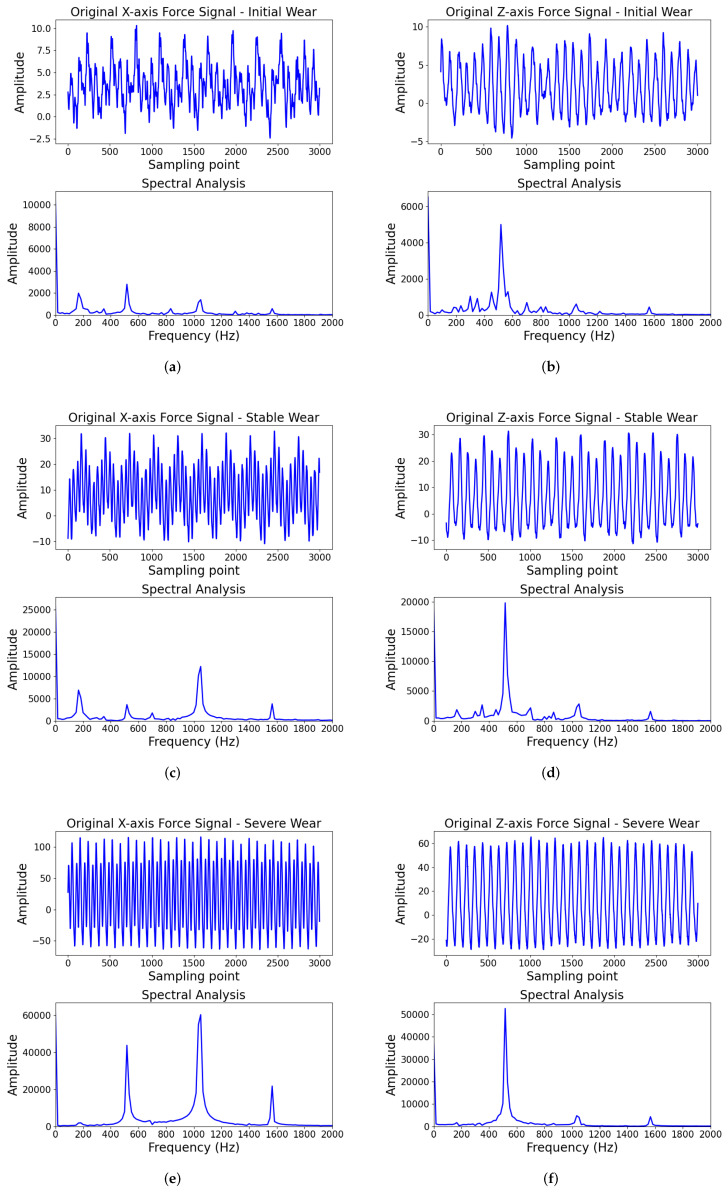
The filtering analysis of force signal. Panels (**a**,**c**,**e**) show x-axis force spectral analysis for initial, stable, and severe wear, respectively, while (**b**,**d**,**f**) show the corresponding z-axis force spectral analysis.

To determine the filtering bands for force and vibration signals, spectral analyses were performed on the three-axis force signals under different wear states, as shown in Figure 11. The effective components of the x- and y-axis forces are mainly concentrated in the low-frequency range and share similar distributions. Therefore, only the x-axis and z-axis spectra are plotted for analysis. As shown in Figure 11a,c,e, the x-axis force signal exhibits pronounced peaks at 150–200 Hz, 500–550 Hz, 1050–1100 Hz, and 1500–1550 Hz, with significant variations in peak amplitudes across different wear states. As illustrated in Figure 11b,d,f, the z-axis force signal shows a prominent peak in the 500–650 Hz range, while minor peak variations are still present around 1500–1550 Hz across different wear stages. During milling, tool wear induces changes in the force signal at the tooth passing frequency and its harmonics, which typically fall within the low-frequency band. Given that both x-axis and z-axis force signals contain effective components in the 1500–1550 Hz frequency range, and this band may contain feature information related to wear states, the low-pass cutoff frequency for the x-, y-, and z-axis force signals is uniformly set to 1600 Hz to ensure complete retention of these effective signal components while suppressing noise in higher frequency bands.

**Figure 12:**
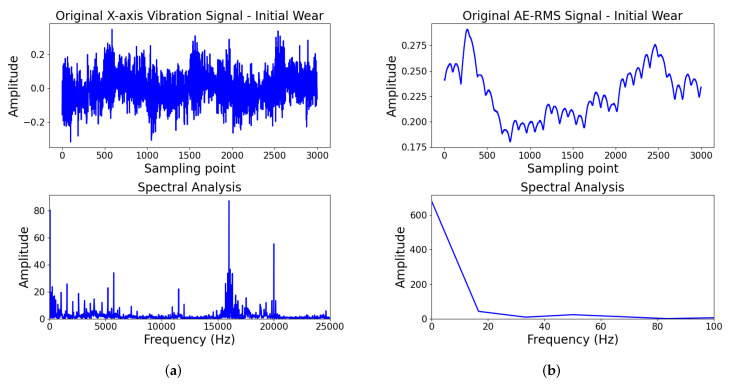

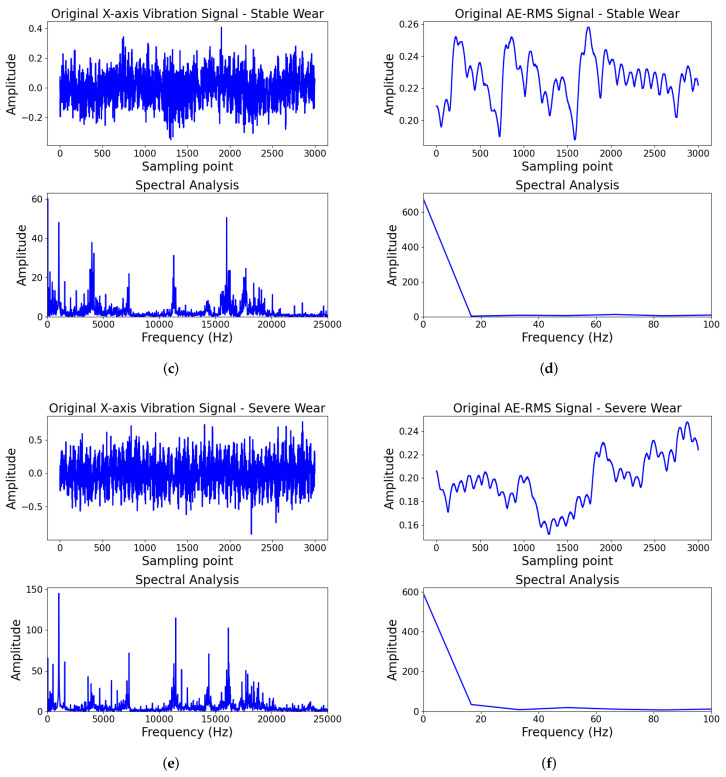
The filtering analysis of vibration and AE-RMS signal. Panels (**a**,**c**,**e**) show x-axis vibration spectral analysis for initial, stable, and severe wear, respectively, while (**b**,**d**,**f**) show the AE-RMS spectral analysis.

Similarly, spectral analyses of the three-axis vibration signals under different wear states show comparable distributions across x, y, and z axes, as shown in Figure 12. In the initial wear state, as shown in Figure 12a, the x-axis vibration exhibits prominent peaks at 2000 Hz and 4000 Hz, with effective components primarily below 5000 Hz. In the stable wear state, as illustrated in of Figure 12c, major peaks cluster near 2000–2500 Hz and 4000–4500 Hz, with effective components similarly concentrated below 5000 Hz. In the severe wear state, as shown in Figure 12e, the x-axis vibration signal exhibits the most prominent peak around 150–200 Hz, with marked peaks also appearing at 500–550 Hz, 1050–1100 Hz, and 1500–1550 Hz, with energy primarily concentrated below 2000 Hz. Although energy is present in the 15,000–20,000 Hz range, its pattern shows no significant differences across wear states and likely originates from machine tool structural vibrations or electromagnetic interference, contributing little to wear identification. During milling, tool wear-related vibration characteristics are primarily reflected at the tooth passing frequency and its harmonics, which typically fall within the mid-to-low frequency range. Given that signals below 10 Hz typically contain environmental noise, DC drift, and low-frequency machine vibrations unrelated to tool wear, a band-pass filter range of 10–5000 Hz is applied to the x-, y-, and z-axis vibration signals to retain effective frequency components related to wear states while suppressing low-frequency drift and high-frequency noise, thereby improving the signal-to-noise ratio and the reliability of feature extraction. In contrast, AE signals exhibit energy-type waveforms in the time domain with small amplitudes and relatively smooth variations, showing no significant low-frequency drift or broadband high-frequency noise, as illustrated of Figure 12b,d,f. Their temporal evolution primarily reflects the cumulative energy from microcrack initiation and friction processes at the tool-workpiece contact interface, rather than sharp transient pulses. Therefore, no additional filtering is applied to the AE channel; only normalization is performed, and the same time window strategy used for other sensors is employed for segmentation. The Bi-TCN convolutional module and channel attention automatically suppress residual noise and extract wear-sensitive AE energy evolution features in a data-driven manner.

After multiple experimental verifications, it was found that dividing the 50,000 sensor data into 50 segments, each with 1000 data points for feature extraction, best reflects the tool wear evolution process at the current stage.

### 5.3. Results Discussion

**Figure 13:**
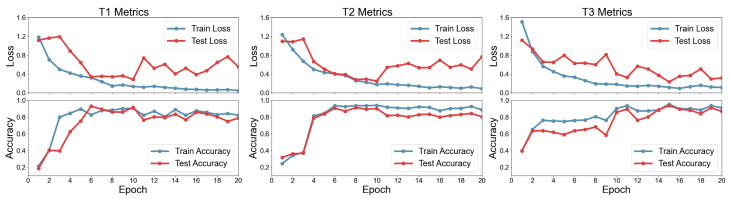
Loss and accuracy curves for training and testing on T dataset.

The training host uses the Win10 operating system, and the training environment is based on PyTorch 12.1. The model was trained on an NVIDIA RTX4050 GPU, with CUDA version 11.8. The training loss adopts cross-entropy loss, and the Adam optimizer is used for model parameter optimization. The optimizer learning rate is set to 0.001, the weight decay is set to 0.00001, and the dropout is set to 0.2 to reduce network overfitting. A mini-batch training strategy is adopted, with the mini-batch size set to 20. The loss curves and accuracy curves during the training process are shown in Figure 13.

On the T1 dataset, the training and testing losses decreased synchronously in the early stages, and the model exhibited strong generalization ability. However, after epoch 7, the testing curve fluctuated, indicating that the model began to partially memorize detailed features of the training data, but still retained a certain degree of generalization. By epoch 6, the model learned more generalizable feature representations through parameter fine-tuning, resulting in a peak test accuracy of 93.02%, at which point the generalization ability was optimal.

On the T2 dataset, due to the significant differences in wear characteristics between C1 and C6 at different stages, the training accuracy increased rapidly from 24.44% to 94.13% in the first 10 epochs, while the test accuracy rose from 31.75% to 90.16%, with both metrics increasing synchronously, demonstrating the model’s effective learning of features from different tool wear states. The 8th epoch achieved the best test accuracy of 91.43%.

Similarly, on the T3 dataset, due to the significant differences in wear characteristics between C1 and C6 at different stages, the training accuracy increased rapidly from 39.68% to 90.16% in the first 10 epochs, while the test accuracy rose from 39.68% to 85.71%, with both metrics increasing synchronously, demonstrating the model’s effective learning of features from different tool wear states. The 15th epoch achieved the best test accuracy of 93.65%. All experiments were performed on GPU, with a total training time of approximately 42 min for 20 epochs and a peak GPU memory usage of 1.6 GB. To evaluate the real-time performance of the model, we measured the inference time on the test sets. The model achieved an average per-sample inference time of 4 ms and a throughput of 270 samples per second, demonstrating that the model can meet the requirements for real-time monitoring applications. In addition, we compared the proposed model with some classical model methods, and the comparison results are shown in Table 4.

The experimental results show that the proposed model demonstrates superior performance on the three cross-validation datasets. Compared with traditional recurrent neural networks such as GRU and LSTM, the proposed model achieves significantly higher classification accuracy on all test datasets. Compared with 1D-CNN-based methods, the proposed model also shows a clear advantage. Compared with 1D-CNN with DGCCA, the proposed model improves the accuracy by 4.48%, 0.63%, and 2.85% on the T1, T2, and T3 datasets, respectively. Compared with CaAt-ResNet-1d, the proposed model achieves accuracy improvements of 6.48%, 1.20%, and 4.15% on the three datasets, respectively. Compared with the latest Transformer architectures, the proposed model improves the accuracy by 3.00% on the T1 dataset, and by 6.37% and 4.13% on the T2 and T3 datasets, respectively. Compared with CNN-Transformer, the proposed model achieves accuracy improvements of 3.50%, 4.89%, and 3.61% on the three datasets, respectively. Compared with Informer, the proposed model achieves accuracy improvements of 3.00%, 1.51% and 2.13% on the T1, T2 and T3 datasets, respectively, and achieves a higher average accuracy. Considering the average accuracy across the three datasets, the proposed model achieves the best accuracy of 92.70% with a standard deviation of 0.93%, verifying its effectiveness in the tool wear classification task.

To verify the contribution of each module to the model performance, four models were designed for ablation experiments. Model 1 removed the channel attention module; Model 2 replaced the Bi-TCN with TCN while retaining the channel attention module; Model 3 replaced Bi-TCN with TCN and simultaneously removed the channel attention module; The Proposed model represents the complete architecture incorporating all designed modules.

The experimental evaluation was conducted on three datasets, and the results are summarized in Table 5. Model 1 achieved an average accuracy of 90.26% ± 0.65%, which is 2.44% lower than the complete model. Model 2 achieved an average accuracy of 90.74% ± 1.77%, which is 1.96% lower than the complete model. Model 3 achieved an average accuracy of 85.50% ± 0.54%, which is 7.20% lower than the complete model. The poor performance is primarily attributed to the model’s lack of adaptive inter-channel weight learning and bidirectional temporal modeling capabilities, resulting in degraded multi-sensor feature fusion and temporal information extraction. The complete model achieved the best performance with an average accuracy of 92.70% ± 0.93%.

The results indicate that both channel attention and Bi-TCN contribute significantly to the model performance. Removing channel attention resulted in a 2.44% accuracy drop; replacing Bi-TCN with TCN resulted in a 1.96% accuracy drop; simultaneously removing both modules caused a 7.20% accuracy drop, demonstrating that both channel attention and Bi-TCN contribute to the performance.

**Figure 14:**
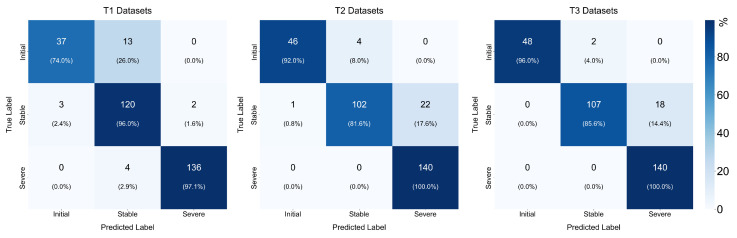
Confusion matrix of experimental results on T dataset.

To further explore the performance of the model, four evaluation metrics of the confusion matrix are used for quantitative analysis: accuracy, precision, recall, and specificity. Accuracy represents the overall classification ability of the model in all tool wear stages, precision reflects the correctness of recognition for each class, recall measures the ability to identify the positive class, and specificity quantifies the misclassification rate. In tool wear monitoring, precision should be prioritized in the initial wear and stable wear stages to avoid unnecessary tool replacement, while in the severe wear stage, more attention is paid to recall and specificity to ensure timely detection of potential failures and prevent a decline in machining quality. The confusion matrices for each T dataset are shown in Figure 14, where the accuracy for all stages, precision for the initial and stable wear stages, recall and specificity for the severe wear stage are shown in Table 6.

It can be seen that in the initial and stable wear stages, the average precision of the three datasets reached 96.8% and 94.0%, respectively, while in the severe wear stage, the average recall and specificity reached 99.1% and 92.0%, respectively, indicating that the model is highly effective in identifying the severe tool wear stage. Through cross-dataset comparative experiments and validation with confusion matrices, the proposed model demonstrates strong feature representation capability and generalization adaptability, accurately identifying tool wear evolution patterns. With a lightweight architecture, it achieves real-time, robust, and high-precision condition monitoring without introducing the frequency domain.

## 6. Conclusions

This work introduces a hierarchical multi-scale dilated temporal convolutional network (Hi-MDTCN) for real-time tool wear monitoring from multi-sensor time series. Within segments, a lightweight channel attention module adaptively amplifies key sensor features; across segments, a bidirectional TCN captures both short- and long-range temporal dependencies, balancing speed and accuracy. Using Butterworth-filtered time-domain inputs only, the approach eliminates extra frequency or time–frequency transforms and reduces preprocessing latency. Experiments show that Hi-MDTCN outperforms baselines in both real-time performance and wear-detection accuracy, offering a reliable solution for tool condition monitoring and intelligent manufacturing.

In future work, we will improve the generalization of the model in diverse tool types and challenging machining conditions, with a focus on wet milling to better reflect industrial practice. Because coolant is commonly used to improve cutting stability, we plan to collect milling data with coolant and, where appropriate, apply transfer learning and domain adaptation to validate robustness in representative shop-floor settings. We will also systematically assess the model’s ability to recognize tool-wear states under varying sampling rates, providing guidance for optimizing sensing and sampling strategies.

## Figures and Tables

**Table 1:** Experimental conditions for PHM 2010.

Equipment Type	Experimental Equipment	Experimental Projects	Parameters
CNC milling machine	Roders Tech RFM760	Spindle speed (r/min)	10,400
Tool type	3-tooth ball nose milling cutter	Feed rate (mm/min)	1555
Workpiece material	Stainless steel HRC52	Cutting width (mm)	0.125
Force sensor	Kistler 9265B dynamometer	Cutting depth (mm)	0.2
Charge amplifier	Kistler 5019A charge amplifier	Tool feeding amount (mm)	0.001
Vibration sensor	Kistler 8636c acceleration sensor	Sampling frequency (kHz)	50
AE sensor	Kistler acoustic emission sensor	Milling method	Climb milling
Wear measuring device	LEICA MZ12 microscope	Cooling method	Dry cutting
Data acquisition card	NI DAQ data acquisition card		

**Table 2:** Cross-validated tool set distribution for T dataset.

Datasets	Training Datasets	Test Datasets
T1	C1–C4	C6
T2	C4–C6	C1
T3	C1–C6	C4

**Table 3:** Hi-MDTCN Model Architecture Parameter Table.

No.	Stage	Module Name	Input Shape	Output Shape	Input Channels	Output Channels	Kernel Size	Dilation
1	Signal Preprocessing	Lowpass Filter (Force)	[N, 3]	[N, 3]	-	-	-	-
2		Bandpass Filter (Vibration)	[N, 3]	[N, 3]	-	-	-	-
3		None Filter (AE)	[N, 1]	[N, 1]	-	-	-	-
4		Segmentation	[50,000, 7]	[50, 7, 1000]	-	-	-	-
5	Force Signal Branch	Conv1d Block	[B × 50, 3, 1000]	[B × 50, 32, 1000]	3	32	3	1
6		Channel Attention	[B × 50, 32, 1000]	[B × 50, 32, 1000]	32	32	3	-
7		MaxPool	[B × 50, 32, 1000]	[B × 50, 32, 1]	32	32	-	-
8	Vibration Signal Branch	Conv1d Block	[B × 50, 3, 1000]	[B × 50, 32, 1000]	3	32	3	1
9		Channel Attention	[B × 50, 32, 1000]	[B × 50, 32, 1000]	32	32	3	-
10		MaxPool	[B × 50, 32, 1000]	[B × 50, 32, 1]	32	32	-	-
11	Acoustic Emission Branch	Conv1d Block	[B × 50, 1, 1000]	[B × 50, 32, 1000]	1	32	3	1
12		MaxPool	[B × 50, 32, 1000]	[B × 50, 32, 1]	32	32	-	-
13	Segment Feature Fusion	Concatenation	[B × 50, 32, 1] × 3	[B × 50, 96, 1]	96	96	-	-
14		Conv1d Block #1	[B × 50, 96, 1]	[B × 50, 32, 1]	96	32	3	1
15		Channel Attention #1	[B × 50, 32, 1]	[B × 50, 32, 1]	32	32	3	-
16		Conv1d Block #2	[B × 50, 32, 1]	[B × 50, 32, 1]	32	32	3	1
17		Channel Attention #2	[B × 50, 32, 1]	[B × 50, 32, 1]	32	32	3	-
18		Reshape to Segments	[B × 50, 32, 1]	[B, 32, 50]	32	32	-	-
19	Segment Temporal Modeling	ResidualDCBlock (Dilation = 1)	[B, 32, 50]	[B, 32, 50]	32	32	3	1
20		ResidualDCBlock (Dilation = 2)	[B, 32, 50]	[B, 32, 50]	32	32	3	2
21		ResidualDCBlock (Dilation = 4)	[B, 32, 50]	[B, 32, 50]	32	32	3	4
22		MaxPool	[B, 32, 50]	[B, 32, 1]	32	32	-	-
23		Fully Connected	[B, 32]	[B, 3]	32	3	-	-

**Note:** B: batch size—20; N: signal length—50.

**Table 4:** Comparison of the accuracy of the experimental results.

Models	T1 (%)	T2 (%)	T3 (%)	Mean ± Std (%)
1D-CNN with DGCCA [39]	88.54	90.80	90.80	90.05 ± 1.30
CaAt-ResNet-1d [40]	86.54	90.23	89.50	88.76 ± 1.95
Informer [48]	90.02	89.92	91.52	90.49 ± 0.89
CNN-Transformer [49]	89.52	86.54	90.04	88.70 ± 1.89
Transformer [50]	90.02	85.06	89.52	88.20 ± 2.73
GRU [51]	83.63	85.72	84.81	84.72 ± 1.05
LSTM [52]	83.52	82.49	81.62	82.54 ± 0.95
GHCRBM [53]	87.81	90.92	62.23	80.32 ± 15.75
**Proposed model**	**93.02**	**91.43**	**93.65**	**92.70 ± 0.93**

**Table 5:** Ablation experiment results.

Model Description	T1 (%)	T2 (%)	T3 (%)	Mean ± Std (%)
Model 1 (without channel attention)	89.52	90.16	91.11	90.26 ± 0.65
Model 2 (TCN instead Bi-TCN with channel attention)	91.75	92.23	88.25	90.74 ± 1.77
Model 3 (TCN instead Bi-TCN without channel attention)	86.03	84.76	85.71	85.50 ± 0.54
**Proposed model**	**93.02**	**91.43**	**93.65**	**92.70 ± 0.93**

**Table 6:** Evaluation indicators on the T dataset.

Indicators	Stage	T1	T2	T3	Average
Accuracy	-	0.9302	0.9143	0.9365	0.9270
Precision	Initial	0.9250	0.9787	1.0000	0.9679
	Stable	0.8759	0.9623	0.9817	0.9399
Recall	Severe	0.9714	1.0000	1.0000	0.9905
Specificity	Severe	0.9886	0.8743	0.8971	0.9200

## Data Availability

The datasets used and/or analysed during the current study are available from the corresponding author on reasonable request.

## Appendix A

**Algorithm A1** The Proposed Framework for Hi-MDTCN
Require: Raw multi-sensor signal $X\in R^{C\times T}$ Ensure: Predicted tool wear class $\hat{y}$ 1: Smooth force signals using sliding window averaging: $f_{smooth}\left[i\right]=\frac{1}{W}\sum_{j=i-\frac{W-1}{2}}^{i+\frac{W-1}{2}}f_{raw}\left[j\right]$ 2: Calculate first-order difference features: $diff\left[i\right]=f_{smooth}\left[i+1\right]-f_{smooth}\left[i\right]$ 3: Compute adaptive threshold $\theta =\bar{d}+k\cdot \sigma_{\left|d\right|}$ 4: Detect tool retraction points when continuous |diff[i]|>θ with matching sign 5: Extract original signal segments closest to the identified tool retraction points 6: Apply 4th-order Butterworth filters with specific cutoff frequencies for different signals 7: Divide signals into *N* equal-length segments $\left\{X_{1},X_{2},\ldots ,X_{N}\right\}$ for multi-scale analysis 8: **for** i=1 to *N* **do** 9: Extract $X_{i}^{\left(\mathrm{dyn}\right)}$, $X_{i}^{\left(\mathrm{acc}\right)}$, $X_{i}^{\left(\mathrm{ae}\right)}$ from segment $X_{i}$ 10: $h_{i}^{\left(\mathrm{dyn}\right)}\leftarrow$ Conv1D + ChannelAttention + MaxPooling on $X_{i}^{\left(\mathrm{dyn}\right)}$ 11: $h_{i}^{\left(\mathrm{acc}\right)}\leftarrow$ Conv1D + ChannelAttention + MaxPooling on $X_{i}^{\left(\mathrm{acc}\right)}$ 12: $h_{i}^{\left(\mathrm{ae}\right)}\leftarrow$ Conv1D + MaxPooling on $X_{i}^{\left(\mathrm{ae}\right)}$ 13: $h_{i}\leftarrow \mathrm{Concat}\left(h_{i}^{\left(\mathrm{dyn}\right)},h_{i}^{\left(\mathrm{acc}\right)},h_{i}^{\left(\mathrm{ae}\right)}\right)$ 14: $f_{i}\leftarrow$ Fusion Conv1D + ChannelAttention on $h_{i}$ 15: **end for** 16: $F\leftarrow [f_{1},f_{2},\ldots ,f_{N}]$ 17: Apply Bi-TCN on *F* 18: z← GlobalMaxPooling over temporal dimension 19: $\hat{y}\leftarrow$ FullyConnected(*z*) 20: **return** $\hat{y}$
